# Left atrial strain analysis improves left ventricular filling pressures non-invasive estimation in the acute phase of Takotsubo syndrome

**DOI:** 10.1093/ehjci/jead045

**Published:** 2023-03-27

**Authors:** Giulia Iannaccone, Francesca Graziani, Marco Giuseppe Del Buono, Massimiliano Camilli, Rosa Lillo, Andrea Caffè, Francesco Moroni, Giulia La Vecchia, Daniela Pedicino, Tommaso Sanna, Carlo Trani, Antonella Lombardo, Gaetano Antonio Lanza, Massimo Massetti, Filippo Crea, Rocco A Montone

**Affiliations:** Department of Cardiovascular and Pulmonary Sciences, Catholic University of the Sacred Heart, Largo Francesco Vito, 1, Rome 00168, Italy; Department of Cardiovascular Medicine, Fondazione Policlinico Universitario A. Gemelli IRCCS, Rome, Italy; Department of Cardiovascular Medicine, Fondazione Policlinico Universitario A. Gemelli IRCCS, Rome, Italy; Department of Cardiovascular and Pulmonary Sciences, Catholic University of the Sacred Heart, Largo Francesco Vito, 1, Rome 00168, Italy; Department of Cardiovascular Medicine, Fondazione Policlinico Universitario A. Gemelli IRCCS, Rome, Italy; Department of Cardiovascular and Pulmonary Sciences, Catholic University of the Sacred Heart, Largo Francesco Vito, 1, Rome 00168, Italy; Department of Cardiovascular Medicine, Fondazione Policlinico Universitario A. Gemelli IRCCS, Rome, Italy; Department of Cardiovascular and Pulmonary Sciences, Catholic University of the Sacred Heart, Largo Francesco Vito, 1, Rome 00168, Italy; Department of Cardiovascular Medicine, Fondazione Policlinico Universitario A. Gemelli IRCCS, Rome, Italy; Department of Cardiovascular and Pulmonary Sciences, Catholic University of the Sacred Heart, Largo Francesco Vito, 1, Rome 00168, Italy; Pauley Heart Center; Wright Center for Clinical and Translational Research, Virginia Commonwealth University, 1200 E Marshall St, Richmond, VA 23298, USA; Department of Cardiovascular and Pulmonary Sciences, Catholic University of the Sacred Heart, Largo Francesco Vito, 1, Rome 00168, Italy; Department of Cardiovascular Medicine, Fondazione Policlinico Universitario A. Gemelli IRCCS, Rome, Italy; Department of Cardiovascular and Pulmonary Sciences, Catholic University of the Sacred Heart, Largo Francesco Vito, 1, Rome 00168, Italy; Department of Cardiovascular Medicine, Fondazione Policlinico Universitario A. Gemelli IRCCS, Rome, Italy; Department of Cardiovascular and Pulmonary Sciences, Catholic University of the Sacred Heart, Largo Francesco Vito, 1, Rome 00168, Italy; Department of Cardiovascular Medicine, Fondazione Policlinico Universitario A. Gemelli IRCCS, Rome, Italy; Department of Cardiovascular and Pulmonary Sciences, Catholic University of the Sacred Heart, Largo Francesco Vito, 1, Rome 00168, Italy; Department of Cardiovascular Medicine, Fondazione Policlinico Universitario A. Gemelli IRCCS, Rome, Italy; Department of Cardiovascular and Pulmonary Sciences, Catholic University of the Sacred Heart, Largo Francesco Vito, 1, Rome 00168, Italy; Department of Cardiovascular Medicine, Fondazione Policlinico Universitario A. Gemelli IRCCS, Rome, Italy; Department of Cardiovascular and Pulmonary Sciences, Catholic University of the Sacred Heart, Largo Francesco Vito, 1, Rome 00168, Italy; Department of Cardiovascular Medicine, Fondazione Policlinico Universitario A. Gemelli IRCCS, Rome, Italy; Department of Cardiovascular and Pulmonary Sciences, Catholic University of the Sacred Heart, Largo Francesco Vito, 1, Rome 00168, Italy; Department of Cardiovascular Medicine, Fondazione Policlinico Universitario A. Gemelli IRCCS, Rome, Italy; Department of Cardiovascular Medicine, Fondazione Policlinico Universitario A. Gemelli IRCCS, Rome, Italy

**Keywords:** LA strain analysis, left ventricular filling pressure, LVEDP, Takotsubo syndrome

## Abstract

**Aims:**

The aim of our study is to assess the ability of left atrial (LA) strain values to improve left ventricular and diastolic pressure (LVEDP) non-invasive estimation as compared with traditional echocardiographic indexes in the acute phase of Takotsubo syndrome (TTS) and to predict adverse in-hospital outcomes in this population.

**Methods and results:**

Consecutive TTS patients were prospectively enrolled. Left ventricular and diastolic pressure was measured at the time of catheterization. Transthoracic echocardiography was performed within 48 h from hospital admission. In-hospital complications (acute heart failure, death from any cause, and life-threatening arrhythmias) were collected. A total of 62 patients were analysed (72.2 ± 10.1 years, female 80%) and in-hospital complications occurred in 25 (40.3%). Left ventricular and diastolic pressure mean value was 24.53 ± 7.92 mmHg. Left atrial reservoir and pump strain values presented higher correlation with LVEDP (*r* −0.859, *P* < 0.001 and *r* −0.848, *P* < 0.001, respectively) in comparison with *E*/*e* ′ ratio, left atrial volume index (LAVi), and tricuspid regurgitation (TR) peak velocity. In addition, at receiver-operating characteristic curve analysis, LA reservoir and pump strain resulted to be better predictors of LVEDP above the mean of our population [0.909 (95% CI 0.818–0.999, *P* < 0.001) and 0.889 (95% CI 0.789–0.988, *P* < 0.001)], respectively] as compared with *E*/*e*′ ratio, LAVi, and TR peak velocity.

Finally, LA reservoir strain resulted to be an independent predictor of worse in-hospital outcomes, together with LVEDP and left ventricular ejection fraction (all *P* < 0.001).

**Conclusion:**

In our study, lower LA reservoir and pump strain values were better predictors of LVEDP as compared with traditional echocardiographic indexes in the acute phase of TTS syndrome. Moreover, LA reservoir strain was an independent predictor of adverse in-hospital outcomes.

## Graphical Abstract

**Graphical Abstract:**
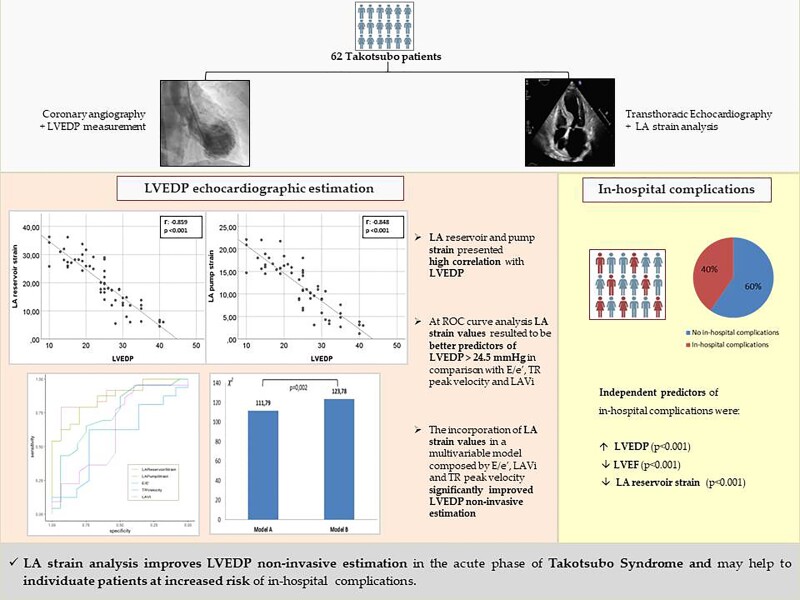
Main results of the study. LVEDP, left ventricular and diastolic pressure; LA, left atrium; ROC, receiving operating characteristic; LAVi, left atrial volume index; TR, tricuspid regurgitation; LVEF, left ventricular ejection fraction.

**See the editorial comment for this article ‘Trouble with estimating filling pressure in acute heart failure: lessons from Takotsubo syndrome’, by O.A. Smiseth, https://doi.org/10.1093/ehjci/jead082.**

## Introduction

Increased left ventricular filling pressure (LVFP) plays a crucial role in heart failure (HF) diagnosis and prognosis.^1^ The gold standard for LVFP assessment is represented by invasive pressure measurement as end-expiratory pulmonary capillary wedge pressure, LV pre-atrial contraction pressure, or left ventricular end diastolic pressure (LVEDP). Elevated LVFP can also be predicted non-invasively by means of standard transthoracic echocardiography through the combination of different traditional echocardiographic indexes, including left atrial volume index (LAVi), tricuspid regurgitation (TR) peak velocity, and *E*/*e*′ ratio.^2–4^ However, this multi-parametric approach is not reliable in the case of missing or contrasting data. Left atrial (LA) function assessment through speckle tracking analysis has been suggested to fill this gap as recent evidence showed that impaired LA strain correlates with increased LVFP in unselected patients referred for non-urgent diagnostic right- or left-sided heart catheterization.^5^ However, there is paucity of studies evaluating the ability of LA strain analysis to improve LVFP non-invasive estimation and, to our knowledge, there are no data as regards patients with acute heart conditions. In the present study, we investigated the linkage between LVFP and LA reservoir and pump strain in the acute phase of Takotsubo syndrome (TTS), as in this homogeneous population of patients, LVEDP shows a wide range of values facilitating the study of the correlation between non-invasive and invasive indexes. Moreover, recent evidence showed that both increased LVEDP and impaired LA strain values are independent predictors of in-hospital complications in patients with TTS.^6–8^ These findings are of utmost importance, as adverse cardiovascular events are not uncommon in this population,^9–11^ and mostly occur within the first days from clinical presentation, thus it is crucial to promptly individuate subjects at increased risk.

Therefore, the aim of our study is to assess whether LA strain values can offer a reliable estimation of LVFP in the acute phase of TTS and to confirm their prognostic power in this population.

## Methods

### Study population

We prospectively enrolled patients presenting with TTS at our hospital (Fondazione Policlinico Universitario Agostino Gemelli IRCCS, Rome). For the present study, we reviewed all cases admitted from January 2020 to November 2021 (*n* = 73). TTS was performed according to the International Takotsubo Diagnostic Criteria.^12^

We excluded from the analysis patients with inadequate echocardiographic acoustic windows (*n* = 6) and cardiac rhythms other than sinus rhythm during the echocardiographic evaluation (*n* = 5).

### Cardiac catheterization

Coronary angiography and ventriculography were performed within 90 min from hospital admission in patients presenting with persistent ST-segment elevation or with hemodynamic or arrhythmic instability, and within 48 h in patients presenting with non-ST-segment elevation. Invasive measurement of LVEDP was performed before LV angiography and after selective coronary angiography.

### Echocardiography

All patients underwent standard 2D echocardiography within 48 h from admission. Images were acquired with patients at rest in the left lateral decubitus position, using commercially available ultrasound systems (Philips Epiq 7: Philips, Amsterdam, The Netherlands) equipped with M5S probe. Two dimensional (2D), colour, pulsed-, and continuous-wave Doppler data were obtained in parasternal and apical views. Sector size, depth, and focus point were adjusted to achieve optimal visualization of all LV and RV myocardial segments at the highest possible frame rate for further analysis. Left ventricular ejection fraction (LVEF) was measured based on the apical 2- and 4-chamber views (A2Ch and A4Ch) using the modified Simpson method.^13^ Left atrial volume was calculated by the biplane disk summation technique in apical four- and two-chamber views, immediately before mitral valve opening and was indexed for body surface area, as suggested by current guidelines.^13^ Tricuspid regurgitation peak velocity was assessed from different echocardiographic views to obtain optimal alignment of continuous-wave Doppler with the TR jet. Left ventricular diastolic function estimation was performed using blood-pooled pulsed Doppler of the mitral valve inflow to measure peak flow velocities in early (*E* wave) and late (*A* wave) systole to quantify the ratio of early-to-late (*E*/*A*) diastolic flow velocity and the deceleration time.^2^ Additionally, Tissue Doppler was used for measuring the ratio between early trans-mitral flow and peak early tissue Doppler velocity (*E*/*e*′) at basal septal level and lateral mitral annulus level from A4Ch view. Left atrial reservoir and pump strain values and LV global longitudinal strain (LV-GLS) were calculated by speckle tracking echocardiography (STE) using frame rates from 40 to 80/s.^14,15^ A standard, commercially available, vendor-independent, dedicated software, 2D Cardiac Performance Analysis© by TomTec-Arena TM (TomTec Imaging Systems, Unterschleissheim, Germany), was used to quantify cardiac wall mechanics. In brief, the endocardial borders of LA and LV were automatically identified over one frame, and endocardial borders were automatically tracked throughout the cardiac cycle. The adequacy of tracking was verified manually, and the region of interest was adjusted to achieve optimal tracking including the entire myocardial wall and to exclude the pericardium. Left atrial strains were measured from A4Chs and LV-GLS from A4Ch, A2Ch and long-axis views^14^ according to the latest consensus. Left atrial reservoir strain was measured from LV-end diastole (mitral valve closure), and LA pump strain was assessed after onset of the *P*-wave in the electrocardiogram. Strain values are reported as absolute numbers throughout the text. All values were obtained from average measurements over three cardiac cycles.

### Assessment of in-hospital complications

In-hospital complications were defined as the composite of acute heart failure (AHF, pulmonary oedema and/or cardiogenic shock; Killip class III/IV), death from any cause, and the occurrence of life-threatening arrhythmias (sustained monomorphic ventricular tachycardia, polymorphic ventricular tachycardia, ventricular fibrillation, high-grade atrioventricular block).

### Statistical analysis

Data distribution was assessed according to the Kolmogorov–Smirnov test. Continuous variables were expressed as mean ± SD or as median (interquartile range). Categorical data were expressed as number (percentage). Correlation analysis was performed to assess the correlation between LVEDP and LA strain, *E*/*e*′ ratio, LAVi, and Peak TR velocity. Moreover, receiver-operating characteristic (ROC) curve analysis was also performed to assess the ability of LA strain, *E*/*e*′ ratio, LAVi, and Peak TR velocity to predict significantly increased LVEDP. The ROC curve analysis was used to estimate the overall predictive accuracy of LVEDP over the mean value of the study population by evaluating the area under the curve (AUC) and the respective 95% confidence interval (CI). Statistically significant differences between the AUCs obtained were evaluated with DeLong and Bootstrap tests as appropriate. Finally, to investigate the incremental predictive power for increased LVEDP non-invasive assessment of LA strain values on the top of standard echocardiographic parameters routinely used, the likelihood ratio test for nested models was performed. The change in global Chi-square was calculated and reported. All analyses were performed using R (The R Foundation) and SPSS (SPSS version 26, Inc., Chicago, IL, USA) statistical softwares. Continuous variables were compared using an unpaired Student’s *t*-test or Mann–Whitney *U* test, and categorical data were evaluated using the Chi^2^ test or Fisher’s exact test, as appropriate. A *P*-value <0.05 was considered statistically significant. Logistic regression analysis for the occurrence of composite in-hospital complications was performed including all variables with a statistically significant *P*-value, separating in different analyses variables with significant multicollinearity, assessed by the variance inflation index (VIF). The study complies with the Declaration of Helsinki and was approved by our Ethics Committee.

## Results

The final study population consisted of 62 patients [mean age 72.2 ± 10.1 years, 50 female (80.6%)]. Mean LVEDP was 24.53 ± 7.92 mmHg. Physical and emotional triggers were the most common underlying triggers (each one occurring in 19 patients, 61.2%); neurological trigger occurred in 3 patients (4.9%), while no evident trigger was identified in 21 patients (33.9%). Apical ballooning type was the most frequent TTS type (55 patients, 91.7%). Non-ST elevation ACS was the most common clinical presentation at admission (41 patients, 67.8%). Left ventricular ejection fraction and LV-GLS were reduced in the majority part of the study population (43.32% ± 11.19 and 14.75 ± 3.95, respectively). Mean values of standard echocardiographic parameters used for non-invasive estimation of LVFP were as follows: LAVi 33.65 ± 11.51 mL/m2, TR peak velocity 2.35 ± 0.57 m/s, *E*/*e*′ ratio 11.94 ± 5.53. The mean LA reservoir and pump strain values were 20.09 ± 8.53 and 11.68 ± 5.46, respectively. Mitral regurgitation (MR) more than mild was detected in 12 patients, with only one case of severe MR (baseline clinical and echocardiographic characteristics of the overall study population are reported in *Table 1*). In-hospital complications occurred in 25 patients (40.3%). In particular, all-cause death occurred in 4 patients (6.4%), with 2 (3.2%) cardiovascular deaths. Acute heart failure occurred in 16 patients (25.8%), while life-threatening arrhythmias in 8 patients (12.9%).

**Table 1:** Baseline patients’ characteristics and comparison between groups with complicated and uncomplicated hospital stay

	Overall population	In-hospital complications	No in-hospital complications	*P*-value (Sig.2-tailed)
	62 pts	25 pts	37 pts	
Baseline clinical characteristics				
Female sex [*n*, (%)]	50 (80.6)	20 (80)	30 (81.1)	0.916
Age (years) [mean ± SD]	72.21 ± 10.07	73.63 ± 10.51	71.3 ± 9.80	0.174
BMI [mean ± SD]	23.89 ± 5.31	23.18 ± 6.46	24.34 ± 4.39	0.528
Hypertension [*n*, (%)]	40 (67.8)	14 (60.9)	26 (72.2)	0.363
Diabetes mellitus [*n*, (%)]	13 (22)	4 (17.4)	9 (25)	0.492
Smoke [*n*, (%)]	20 (32.2)	7 (28)	13 (35)	0.603
Clinical presentation				
Trigger [*n*, (%)]				**0.022**
Class I: no trigger	21 (33.9)	7 (28)	14 (37.8)	
Class IIa: emotional trigger	19 (30.6)	4 (16)	15 (40.5)	
Class IIb: physical trigger	19 (30.6)	13 (52)	6 (16.3)	
Class III: neuro trigger	3 (4.9)	1 (4)	2 (5.4)	
Type of ACS [*n*, (%)]				0.711
NSTEMI	41 (67.8)	16 (64)	24 (68.6)	
STEMI	21 (32.2)	9 (36)	11 (31.4)	
Heart rate (bpm)				0.429
[mean ± SD]	88.29 ± 21.31	89.86 ± 27.27	87.24 ± 16.6	
Coronary angiography				
LVEDP (mmHg)				**<0.001**
[mean ± SD]	24.53 ± 7.92	29.72 ± 7.11	20.47 ± 5.95	
LV-preA (mmHg) [mean ± SD]	16.14 ± 6.24	20.08 ± 5.66	13.06 ± 4.82	**<0.001**
Coronary artery disease [*n*, (%)]				0.553
Normal coronary arteries	19 (30.6)	7 (28)	12 (32.4)	
Angiographic stenosis <50%	31 (50)	12 (48)	19 (51.3)	
Angiographic stenosis >50%	12 (19.4)	6 (24)	6 (16.3)	
Echocardiography				
LV EDV (ml)	87.47 ± 19.32	92.81 ± 22.32	83.97 ± 16.51	0.719
[mean ± SD]				
LV ESV (ml)	49.08 ± 19.34	53.9 ± 25.17	45.59 ± 13.11	0.250
[mean ± SD]				
LVEF (%)	43.32 ± 11.19	38.44 ± 12.76	47.64 ± 9.61	**<0.001**
[mean ± SD]				
LV GLS (%)	14.75 ± 3.95	13.07 ± 3.6	15.69 ± 3.88	0.070
[mean ± SD]				
LAVi (mL/mq)	33.65 ± 11.51	39.34 ± 13.45	30.29 ± 11.26	**0.001**
[mean ± SD]				
LA reservoir strain	20.09 ± 8.53	16.8 ± 7.96	23.76 ± 6.96	**<0.001**
[mean ± SD]				
LA pump strain (%)	11.68 ± 5.46	9.65 ± 5.2	13.87 ± 4.56	**<0.001**
[mean ± SD]				
TR peak velocity (m/s)	2.35 ± 0.57	2.58 ± 0.64	2.2 ± 0.48	0.169
[mean ± SD]				
E/A	0.69 ± 0.30	0.935 ± 0.4	0.819 ± 0.2	0.514
[mean ± SD]				
*E*/*e*′	11.94 ± 5.53	12.17 ± 6.15	11.76 ± 5.14	0.919
[mean ± SD]				
DT (ms)	190.27 ± 71	203.25 ± 90.4	181 ± 53.02	0.368
[mean ± SD]				
TAPSE (mm)	19.47 ± 3.68	19.38 ± 4.28	19.53 ± 3.29	0.767
[mean ± SD]				
PASP (mmHg)	35.07 ± 9.66	37.44 ± 9.02	33.42 ± 9.9	0.077
[mean ± SD]				
RVFAC (%)	37.15 ± 10.5	34.83 ± 10.28	38.61 ± 10.53	0.255
[mean ± SD]				
MR > mild	12 (19.3)	5 (20)	7 (18.9)	0.188
[*n*, (%)]				
AR > mild	4 (6.8)	1 (4.2)	3 (8.6)	0.233
[*n*, (%)]				
TR > mild	11 (18.6)	3 (12.5)	8 (22.9)	0.226
[*n*, (%)]				
Apical type	55 (91.7)	20 (83.3)	35 (97.2)	0.057
[*n*, (%)]				
Mid-ventricular type	5 (8.3)	3 (12.5)	2 (5.6)	0.340
[*n*, (%)]				
Basal type	1 (1.7)	1 (4.2)	0 (0)	0.217
[*n*, (%)]				
Focal type	0	0	0	-
[*n*, (%)]				
Global type	0	0	0	-
[*n*, (%)]				
Laboratory				
Tr I (ng/mL)	5.93 (26.87)	3.7 (43.98)	5.97 (275.7)	0.70
[median (IQR)]				
NT-proBNP (pg/mL)	8427 (13670)	6568 (10654)	8604 (21935)	0.844
[median (IQR)]				
Creatininemia (mg/dL)	0.85 ± 0.26	0.96 ± 0.35	0.77 ± 0.14	0.312
[mean ± SD]				
CRP (mg/dL)	19.5 (35.5)	32.5 (87.3)	18.3 (18.2)	0.178
[median (IQR)]				

Bold represents *P*-value with statistical significance (<0.05).

BMI, body mass index; ACS, acute coronary syndrome; NSTEMI, non-ST elevation myocardial infarction; STEMI, ST-elevation myocardial infarction; LVEDP, left ventricular end diastolic pressure; LV pre-A, left ventricular pre-atrial contraction pressure; LVEDV, left ventricular end diastolic volume; LVEDS, left ventricular end systolic volume; LVEF, left ventricular ejection fraction; LV-GLS, left ventricular global longitudinal strain; LAVi, left atrial volume index; LA, left atrium; TR, tricuspid regurgitation; DT, deceleration time; TAPSE, tricuspid annulus planar systolic excursion; PASP, pulmonary artery systolic pressure; RVFAC, right ventricle fractional area; MR, mitral regurgitation; AR, atrial regurgitation; Tr, troponin.

The Graphical Abstract summarizes the main results of our study.

### Echocardiographic estimation of LVEDP

For both LA reservoir and pump strain, progressively lower strains were associated with increasingly higher LVEDP (*r* −0.859, *P* < 0.001 and *r* −0.848, *P* < 0.001, respectively). Left atrial reservoir and pump strain had stronger correlation with LVEDP than both LAVi and *E*/*e*′ ratio (*r* 0.385, *P* = 0.019 and *r* 0.505, *P* = 0.001, respectively), while peak TR velocity was not significantly correlated with LVEDP (*r* 0.058, *P* = 0.773) (see Supplementary data online, *Figure S1*).

**Figure 1:**
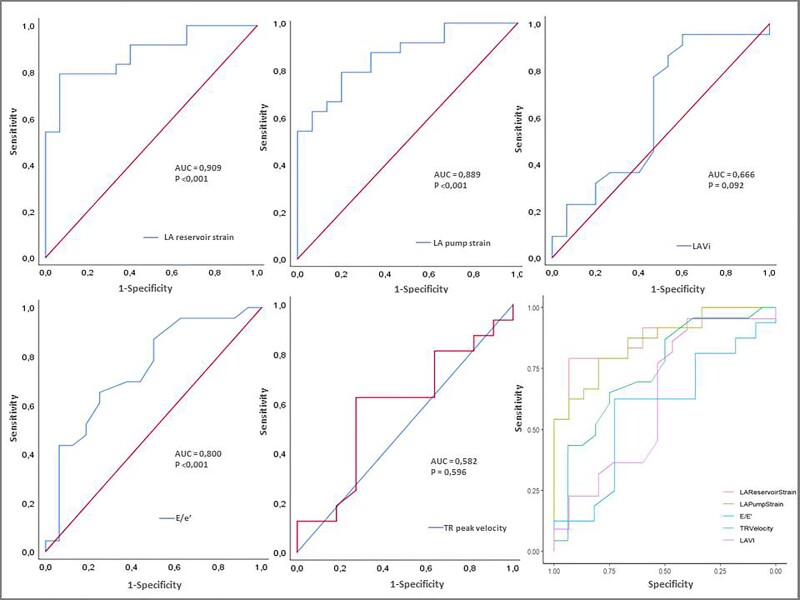
Predictors of increased LVEDP. ROC curve analysis was performed to compare the ability to estimate significantly increased LVEDP (>24.5 mmHg) of LA strain values and standard echocardiographic parameters currently used for non-invasive LVFP assessment. As a result, higher AUCs were obtained for LA reservoir and pump strain in comparison with *E*/*e*′ ratio, even though DeLong analysis showed no significant difference. Conversely, AUCs for LAVi and TR peak velocity were not significant. ROC, receiving operating characteristic; LVEDP, left ventricular and diastolic pressure; LA, left atrium; LVFP, left ventricular filling pressure; AUC, area under the curve; LAVi, left atrial volume index; TR, tricuspid regurgitation.

The ROC curve analysis was performed to investigate the ability of LA strain values and traditional echocardiographic indexes to predict an increase of LVEDP above the mean of our population, whereas >24.5 mmHg (*Figure 1*). Of importance, higher AUCs were obtained for LA reservoir and pump strain [0.909 (95% CI 0.818–0.999, *P* < 0.001) and 0.889 (95% CI 0.789–0.988, *P* < 0.001)], respectively] compared with *E*/*e*′ratio [0.800 (95% CI 0.663–0.937, *P* < 0.001)], LAVi [0.666 (95% CI 0.473–0.859, *P* = 0.092)], and TR peak velocity [0.582 (95% CI 0.35–0.814, *P* = 0.596)]. AUCs for both LA reservoir and pump strain were significantly higher than the AUCs for LAVi (*P* = 0.029 and *P* = 0.048) and for TR peak velocity (*P* = 0.05 in both cases), but not for *E*/*e*′ ratio (*P* = 0.11 and *P* = 0.155) (*Figure 1*).

A cut-off value of 17% for LA reservoir strain and of 9% for LA pump strain yielded the highest sensitivity and specificity for the prediction of LVEDP >24.5 mmHg (respectively 80% and 99% for LA reservoir strain and 79% and 99% for LA pump strain).

**Figure 2:**
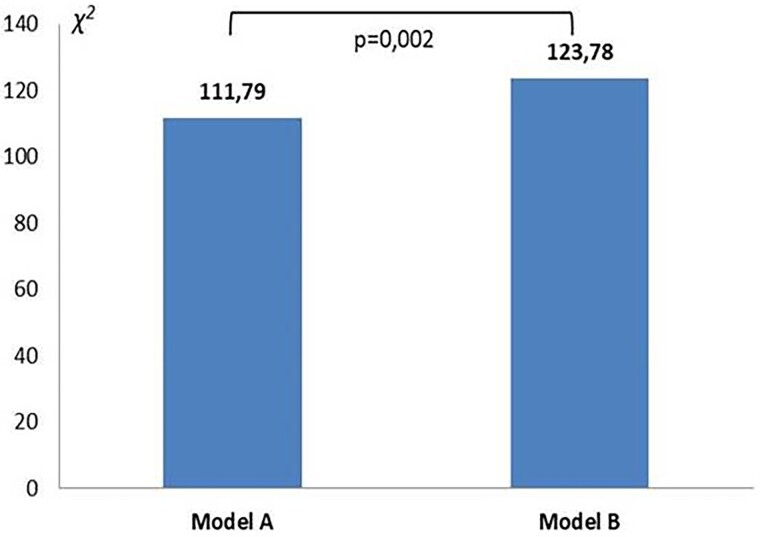
Changes in multivariable model according to LA strain inclusion or exclusion. The incorporation of LA strain values in a multivariable model predictive of LVEDP >24.5 mmHg composed by *E*/*e*′ ratio, LAVi, and TR peak velocity lead to an incremental predictive value (changes in X2 = 11.99; *P* = 0.002). Model A includes only *E*/*e*′ ratio, LAVi, and TR peak velocity, while in model B, LA strain values were incorporated. LA, left atrium; LVEDP, left ventricular and diastolic pressure; LAVi, left atrial volume index; TR, tricuspid regurgitation.

Finally, we observed that in 11 patients (20%), non-invasive estimation of increased LVEDP could not have been performed using the conventional algorithm including *E*/*e*′ ratio, LAVi and Peak TR velocity, due to the lack of more than one parameter or conflicting results. Therefore, we performed a likelihood ratio test which showed that the incorporation of LA strain values in a multivariable model predictive of LVEDP >24.5 mmHg, composed by *E*/*e*′ ratio, LAVi, and TR peak velocity, led to an incremental predictive value (changes in X2 = 11.99; *P* = 0.002) (*Figure 2*).

### Predictors of in-hospital complications

Patients who experienced in-hospital complications had a higher prevalence of physical trigger (*P* = 0.022), higher LVEDP (29.72 ± 7.11 vs. 20.47 ± 5.95, *P* < 0.001), lower LVEF values (*P* < 0.001), larger LAVi (*P* = 0.001), and lower LA reservoir and pump strain (both *P* < 0.001) as compared with those who did not experience in-hospital complications (*Table 1*, Supplementary data online, *Figure S2*).

At univariate logistic regression analysis, higher LVEDP (*P* = 0.003), lower LVEF (*P* = 0.02), LA reservoir strain (*P* = 0.004), and LA pump strain (*P* = 0.039) were predictors of in-hospital complications (*Table 2*). The analysis of the VIF showed significant multicollinearity not only between LA reservoir and pump strain, as expected, but also between LVEDP and LA strain values (VIF >5 in all cases). Conversely, no multicollinearity was detected between LVEF and either LA strain values or LVEDP (VIF < 2 in both cases). Multivariate logistic regression analyses were performed including LVEF and separately LVEDP and LA reservoir strain. As a result, all variables remained independent predictors of in-hospital complications (all *P* < 0.001) (*Table 2*). Left atrial pump strain was excluded in reason of the low predictive power at univariate analysis.

**Table 2:** Univariate and multivariate regression analysis to predict in-hospital complications

	Univariate analysis		Multivariate analysis	
	OR (95% CI)	*P-value*	OR (95% CI)	*P-value*
Physical trigger	0.533 (−0.038–7.487)	0.641	—	—
LVEDP	1.218 (1.071–1.385)	**0.003**	1.36 (1.15–1.62)	**<0.001**
LVEF	0.943 (0.898–0.991)	**0.02**	0.85 (0.77–0.93)	**<0.001**
LAVi	1.037 (−0.994–1.083)	0.091	—	—
LA reservoir strain	0.909 (0.850–0.971)	**0.004**	0.82 (0.73–0.91)	**<0.001**
LA pump strain	0.927 (0.863–0.996)	**0.039**	—	—
Peak TR velocity	3.929 (−0.990–15.590)	0.052	—	—

Bold represents *P*-value with statistical significance (<0.05).

LVEDP, left ventricular end diastolic pressure; LVEF, left ventricular ejection fraction; LVGLS, left ventricular global longitudinal strain; LAVi, left atrial volume index; LA, left atrium; TR, tricuspid regurgitation.

**Figure 3:**
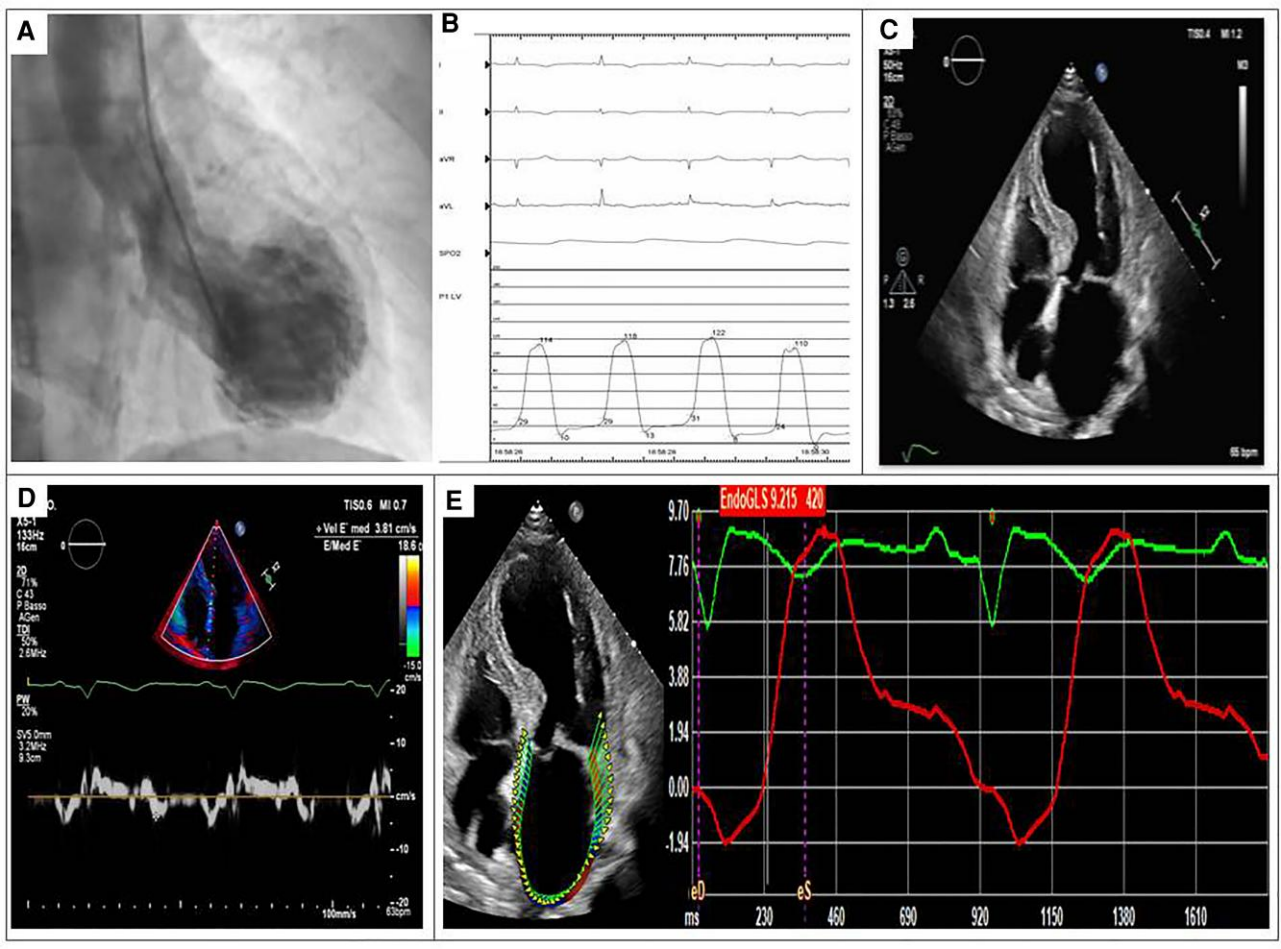
Illustrative example of a TTS patient who experienced complicated in-hospital stay. Panel A: ventriculography showing typical apical ballooning. Panel B: LV pressure trace acquired during left heart catheterization showing high LVEDP. Panel C: echocardiographic A4Ch view confirming the presence of typical apical ballooning and enlarged left atrium. Panel D: echocardiographic measurement of *E*/*e*′ ratio (increased). Panel E: LA speckle tracking analysis showing impaired values of both LA reservoir and pump strain. TTS, Takotsubo; LVEDP, left ventricular and diastolic pressure; A4Ch, apical 4 chamber; TR, tricuspid regurgitation; LA, left atrium.

*Figure 3* illustrates angiographic and echocardiographic images from a patient included in our study who experienced in-hospital complications due to AHF and presented increased LVEDP, impaired LA strain values, and high LAVi, *E*/*e*′ ratio, and TR peak velocity.

## Discussion

To the best of our knowledge, this is the first study to prove the correlation between invasively assessed LVEDP and LA strain values in TTS patients and, broadly speaking, in an acute setting. Left atrial reservoir and pump strain values proved to increase LVEDP non-invasive predictability in TTS patients in our study as compared with traditional indexes including *E*/*e*′ ratio, LAVi, and TR peak velocity. In accordance with our results, a recent study by Dyaco et al.^16^ showed poor correlation between traditional echocardiographic parameters and invasively assessed LVFP in patients with TTS, thus further encouraging the evaluation of additional non-invasive indexes to estimate LV diastolic function in this population.

The reasons of the correlation between LVEDP increase and LA impairment are not completely understood. One possible explanation may be that increased LVFP is associated with reduced LV systolic performance, which may worsen LA function. Another possible explanation is that elevated LVFP may increase LA pressure with consequent augmented LA wall stress. Moreover, increased LVEDP is usually associated with altered LA volumes, which may spill over onto LA mechanics. Moreover, the evidence of multicollinearity at VIF analysis endorses the hypothesis that these variables are indicative of the same phenomenon. This finding is consistent with what was recently assessed by Smiseth et al.^17^ who suggested a novel algorithm for echocardiographic estimation of LVEDP in patients with HF and preserved ejection fraction, which includes LA reservoir strain when one or more conventional parameters are missing and the remaining present conflicting results.

There are few data regarding LA mechanics alterations in TTS, hence LA speckle tracking analysis is not mentioned in the recent joint consensus document on multimodality imaging in TTS.^18^ However, all available studies/case series highlight a transient LA dysfunction in the acute phase of TTS with an almost complete recovery at follow-up, similar to what happens to LV.^7,8,19^ The mechanisms underlying LA dysfunction in this patient population are still unclear. Plausible pathophysiological explanations to this phenomenon include the possible role of the cathecolaminergic activation,^20^ and the impact on atrial walls of the increased LVFP consequent to LV dysfunction.^12^

Speckle tracking analysis of LA performance allows not only to unveil subtle LA function impairment but also to define the phase of LA mechanics, which compromised the most. At present, most studies on LA dysfunction performed in unselected patients in stable clinical conditions showed an equal impairment of all LA functional phases (reservoir, conduit, and pump) in unselected patients.^5^ However, a cardiac magnetic resonance (CMR) study highlighted a transient reduction of LA reservoir function against an initial increase in LA pump performance in TTS, which has been explained as an early compensatory mechanism.^8^ On the other hand, we found an equal reduction of both LA reservoir and pump functions in the present study. However, our analysis was conducted in the very early phase, evaluating echocardiographic images mostly collected within 24 h from hospital admission, while in the study by Backhaus et al., CMR was performed within 3 days since TTS clinical presentation. This temporal difference may suggest that the compensatory mechanism of LA pump function increase could not occur in the very early phase of TTS and/or that medical therapies administered in the first few days can have an impact on LA recovery. In addition, the use of different imaging modalities could account for the dissimilar results. Lastly, as discussed above, LA impairment in TTS has also been related to the catecholamine effects and it is well known that there is a wide inter-personal difference in beta-receptors expression on the LA,^21^ thus discordant results across small patient populations are not surprising.

In reason of the novelty of the technique, LA strain thresholds of abnormality are still debated. Concordantly, with the results obtained by Inoue et al.,^5^ latest recommendations^17^ indicate that values below 18 for LA reservoir strain and 8 for LA pump strain are predictive of LVFP >12 mmHg. These thresholds do not significantly differ from the cut-off values individuated in our study to estimate LVEDP >24.5 mmHg (17 for LA reservoir and 9 for LA pump strain). The explanation to this apparent discrepancy is that Inoue et al. mainly used pulmonary capillary wedge pressure and LV pre-atrial contraction pressure to assess LVFP, while we performed our analysis considering exclusively LVEDP. In fact, LVEDP may be up to 5–10 mmHg higher than the above mentioned invasive indexes, as Dayco at al.^16^ reported in their TTS population and we confirmed in ours.

Another relevant finding of our study is that impaired LA reservoir strain resulted to be an independent predictor of in-hospital complications in this population, confirming previous data.^7,8^

Left atrial function impairment represents a well-known predictor of adverse cardiovascular events in patients suffering from heart disease of various aetiologies.^22–24^ Indeed, LA dysfunction is considered a hallmark of global myocardial damage related to several pathological processes, including LV systolic and diastolic dysfunction, heart failure, LA fibrosis, atrial fibrillation, and reduced exercise tolerance.^25–28^

Larger studies are warranted to ascertain whether LA speckle tracking analysis may contribute to identify TTS patients at higher risk of worse in-hospital outcomes.

### Limitations

Some limitations of the present study should be acknowledged. Firstly, the small sample size with a relative low number of events could have influenced the results of our analysis. However, the clinical characteristics and outcomes of our patients are similar to those encountered in larger studies, thus making our population well representative of TTS patients. Moreover, we did not consider a control group as LVEDP measurement is not routinely assessed during cardiac catheterization unless in case of high suspicion for TTS or significant left ventricular diastolic dysfunction. Likewise, echocardiographic follow-up data are not available, but the transitory nature of LA impairment in TTS patients has already been acknowledged in previous studies^7,8,19^ and invasive LVEDP measurement is obviously not routinely performed at follow-up. In addition, larger studies including patients with acute LVFP increase of different aetiologies are required to confirm the ability of LA speckle tracking analysis to predict sudden LVEDP impairment.

## Conclusions

In the acute phase of TTS, impaired LA strain values correlate with increased LVEDP better than traditional indexes including *E*/*e*′ ratio, LAVi, and TR peak velocity. Moreover, LA reservoir strain resulted to be an independent predictor of adverse in-hospital outcomes in this population. Left atrial strain analysis may also improve LVFP non-invasive estimation in acute clinical settings and may help to individuate patients at increased risk of in-hospital complications.

## Supplementary data

Supplementary data is available at *European Heart Journal - Cardiovascular Imaging* online.

## Supplementary Material

jead045_Supplementary_Data

## Data Availability

The data underlying this article are available in the article and in its online supplementary material.
