# Supplementary material for: Left atrial strain analysis improves left ventricular filling pressures non-invasive estimation in the acute phase of Takotsubo syndrome
Source: Eur Heart J Cardiovasc Imaging. 2023 Mar 27;24(6):699–707. doi: 10.1093/ehjci/jead045 (PMC10274307; doi:10.1093/ehjci/jead045)
Supplement: jead045_Supplementary_Data [file jead045_supplementary_data.docx]

**Supplementary materials**.

**Figure S1:** Correlation analysis.


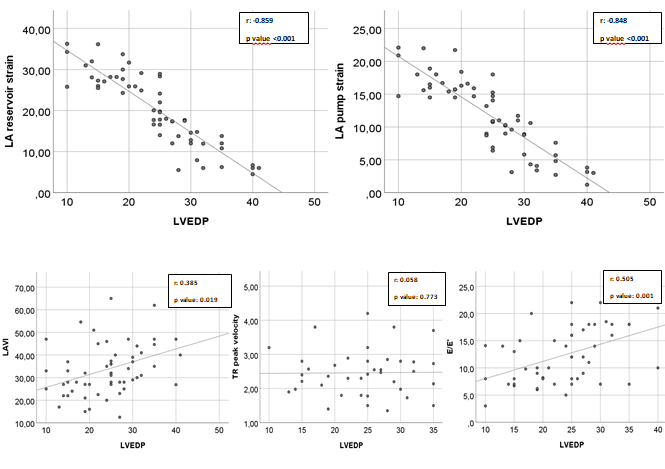


*Legend:* At correlation analysis LVEDP was inversely correlated with both LA reservoir and pump and directly correlated E/e’ ratio and LAVi, even though lower correlation indexes were detected for the latters. Conversely, no correlation was found between LVEDP and TR peak velocity.

*Abbreviations:* LVEDP: left ventricular and diastolic pressure; LA: left atrium; LAVi: left atrial volume indexed; TR: tricuspid regurgitation.

**Figure S2:** Box plot patients with in-hospital complications vs those without.


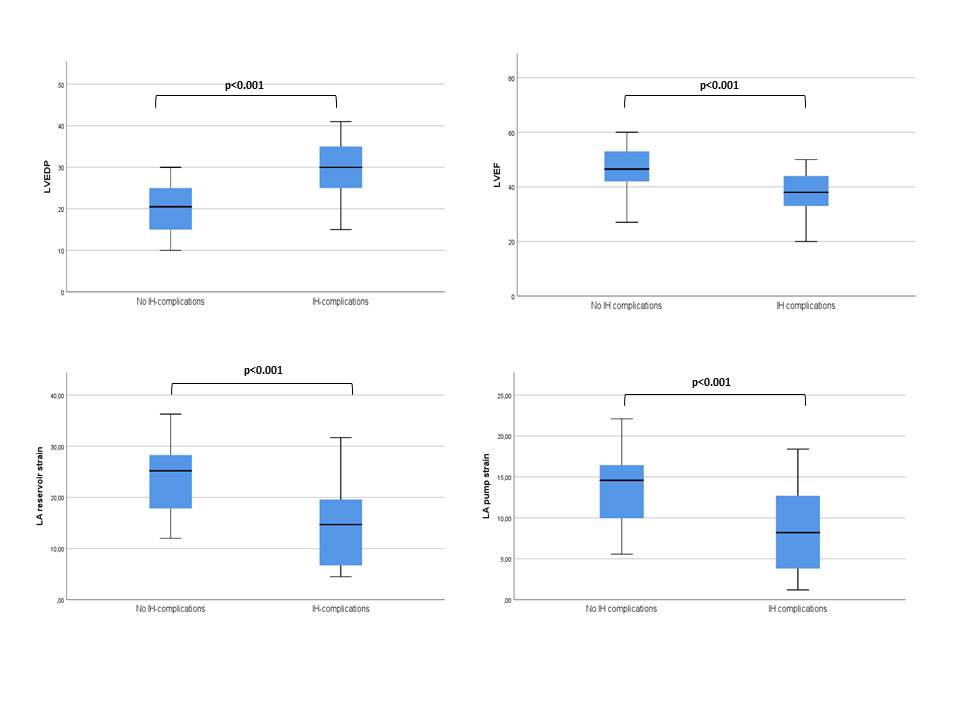


*Legend:* Box plot with median and interquartile ranges of LVEDP, LVEF, LA reservoir and pump strain in patients with complicated vs uncomplicated in-hospital stay.

*Abbreviations:* LVEDP: left ventricular and diastolic pressure; LVEF: left ventricular ejection fraction; LA: left atrium.
